# Tropical forest conversion to rubber plantation affects soil micro- & mesofaunal community & diversity

**DOI:** 10.1038/s41598-019-42333-4

**Published:** 2019-04-10

**Authors:** Dharmesh Singh, J. W. Ferry Slik, Yoon-Seong Jeon, Kyle W. Tomlinson, Xiaodong Yang, Jin Wang, Dorsaf Kerfahi, Dorota L. Porazinska, Jonathan M. Adams

**Affiliations:** 1 Center for Integrative Conservation, Xishuangbanna Tropical Botanical Garden, Chinese Academy of Sciences, Menglun, Mengla, Yunnan, 666303 China; 2 Environmental Biotechnology & Genomics Division, CSIR-NEERI, Nehru Marg, Nagpur, MH 440020 India; 3 Faculty of Science, Universiti Brunei Darussalam, Jalan Tungku, BE1410 Brunei Darussalam; 4 ChunLab Inc., Bldg. 105-1, Suite #307, Seoul National University, 1 Gwanak-ro, Gwanak-gu, Seoul, 151-742 Republic of Korea; 5 CAS Key Laboratory of Tropical Forest Ecology, Xishuangbanna Tropical Botanical Garden, Chinese Academy of Sciences, Menglun, Mengla, Yunnan, 666303 China; 6 Department of Biological Sciences, College of Natural Sciences, Seoul National University, Gwanak-Gu, Seoul, 151-742 Republic of Korea; 7 Department of Entomology and Nematology, University of Florida, IFAS, 1881 Natural Area Drive, Gainesville, 32611 Florida USA; 8 School of Geography and Ocean Sciences, Nanjing University, Nanjing, 210023 Jiangsu Province China

## Abstract

Tropical rainforests play important roles in carbon sequestration and are hot spots for biodiversity. Tropical forests are being replaced by rubber (*Hevea brasiliensis*) plantations, causing widespread concern of a crash in biodiversity. Such changes in aboveground vegetation might have stronger impacts on belowground biodiversity. We studied tropical rainforest fragments and derived rubber plantations at a network of sites in Xishuangbanna, China, hypothesizing a major decrease in diversity with conversion to plantations. We used metabarcoding of the 18S rRNA gene and recovered 2313 OTUs, with a total of 449 OTUs shared between the two land-use types. The most abundant phyla detected were Annelida (66.4% reads) followed by arthropods (15.5% reads) and nematodes (8.9% reads). Of these, only annelids were significantly more abundant in rubber plantation. Taken together, α- and β-diversity were significantly higher in forest than rubber plantation. Soil pH and spatial distance explained a significant portion of the variability in phylogenetic community structure for both land-use types. Community assembly was primarily influenced by stochastic processes. Overall it appears that forest replacement by rubber plantation results in an overall loss and extensive replacement of soil micro- and mesofaunal biodiversity, which should be regarded as an additional aspect of the impact of forest conversion.

## Introduction

Tropical rainforests play important roles in biogeochemical cycling and climate regulation and act as reservoirs of global biodiversity by supporting around 50% of all described species^1–3^. Native forest areas are shrinking throughout the tropics, with rapid clearance for agriculture and plantations^2,4–6^. More than one million hectares of native forest have been converted to rubber plantation over the last few decades in the Mekong River Region alone^7^. This has led to a substantial reduction in structural and functional biodiversity^5,8–10^, a reduction in carbon sequestration^11,12^, and an increase in habitat fragmentation^5,13^ with noticeable alterations in the hydrological systems^14–16^. Forest conversion into plantations also drives changes in soil chemistry, for instance in pH, C: N ratio, P and Ca concentrations^17,18^. The resulting chemical degradation of tropical soils is fast and often results in a complete or partial loss of its productive capacity^19^.

Anthropogenic alteration of forests, through logging or replacement of native logged forest by plantation (which generally uses introduced species), may have various effects on alpha (α) and beta (β) diversity of the plants or animals being studied, but most of the studies suggest a decline in both measures of diversity^3,5,9^. In contrast to larger organisms, bacteria have been shown to undergo both an increase^20^ as well as a decrease^21^ in β-diversity following forest conversion to agriculture, with an increase or no effects on α-diversity^20–22^.

Soil microfauna (roughly defined as 5–120 µm) and mesofauna (somewhat inconsistently defined as 80 µm–2 mm), includes adults of various phyla (e.g., Nematoda, Tardigrada, Rotifera, small Annelida), and eggs/juvenile stages of some metazoan species^23^. These organisms have been often neglected from soil biodiversity surveys even though they are considered to play an important role in land ecosystem functioning. For example, nematodes play important roles in soil ecological processes such as nutrient cycling and plant growth^24,25^ and are sensitive enough to environmental changes to be used as bioindicators for ecotoxicological assessment of soil^26^.

In the past few years, DNA sequencing-based analyses of soil and sediment micro- and mesofaunal communities have revealed divergent lineages^27–29^, accompanied with large datasets of unknown sequences within known phyla that hint at a vast but poorly known diversity. Amongst Nematoda, for instance, it has been estimated that <4% of total species diversity is formally described^30^. This information gap is principally due to difficulty in studying these organisms because of their small size and hard-to-distinguish morphology at the species taxonomic level.

In this study, we focus on rainforests in the global biodiversity hotspot of the Xishuangbanna region of Yunnan, SW China. In Xishuangbanna, the original primary forest, as well as the derived logged forest, has been converted rapidly to rubber (*Hevea brasiliensis* Müll. Arg.) plantation. In 1992, rubber covered 87,000 ha, rising to 153,000 ha in 2002 and 424,000 ha in 2012^16^. The impact of forest conversion to rubber plantation in southern China has already been explored with respect to soil nematodes, which is one aspect of micro- and mesofaunal diversity. Using traditional morphological methods, Xiao *et al*.^31^ compared nematode diversity in natural forests and rubber plantations, finding that conversion was associated with a decrease in both α- and β- diversity.

The present study goes further in encompassing the soil micro- and mesofauna and by including a larger number of sites than was sampled by Xiao *et al*.^31^. The present study also utilizes high-throughput sequencing interpreted with the help of biologically informative taxonomic resources covering alternate loci such as the 18S rRNA gene^32^. As such the technique is capable of recognizing cryptic biodiversity even within relatively well-studied groups (e.g., Nematoda), as well as undocumented biodiversity in relatively poorly studied groups (e.g., Tardigrada).

Our main hypotheses in this study were as follows:

1. We expected that forest conversion to rubber plantation would significantly alter the soil community composition, with each land-use type harboring a distinct soil metazoan community, with the forest conversion driving changes in soil chemistry which in turn modified the soil biota.
2. We predicted lower soil micro- and mesofaunal α- diversity in rubber plantation.
3. We also predicted lower β- diversity of micro- & mesofauna within rubber plantation compared to the native forest.
4. We predicted that the micro- & mesofaunal community assembly in both the native forest and derived rubber plantations would be strongly influenced by deterministic rather than stochastic/neutral processes, just as is the case for soil and sediment microbes from similar environments^33–35^.

## Materials and Methods

### Study location

The study was conducted around the Xishuangbanna Tropical Botanical Garden, XTBG (21°09′–22°33′N, 99°58′–101°34′E), in Xishuangbanna, Yunnan, China, concentrating on primary natural rainforests and adjacent monoculture rubber plantations. The mean annual temperature (MAT) and mean annual precipitation (MAP) were 21.7 °C and 1512 mm (1980–2008), respectively^36^.

### Study area description

Xishuangbanna has a seasonal outer tropical climate, with hot, rainy summers and somewhat cooler and drier winters, which remain free of frost^36^. The tropical rainforests of Xishuangbanna are characterized by high species richness with up to 4180 species of vascular plants/2500 m^2^ plot and prevalence of epiphytes and epiphyllous mosses on the trees with the ubiquitous presence of woody lianas and strangling plants^37^. The tropical rainforest can be classified into two subtypes: tropical seasonal rain forest (in the lowlands; <900 m a.s.l., or meters above sea level) and tropical montane rain forest (900–1600 m a.s.l.)^38^, but with soil type differentiation within each of these (for soil types see Supplementary Table S1). For example, some areas of the lowland and montane forest are on limestone soils, with a distinctive plant community.

### Field sampling

Sampling was carried out during the month of August 2013. Seven rainforest sampling sites were chosen within a 10 km radius of XTBG. Seven rubber plantation sites were chosen adjacent to each old-growth forest sampling site. In this study, there are fourteen sites in total (seven forests and seven rubber). At each sampling site, four quadrats (10 m × 10 m), were located >30 m apart along a linear transect. See SI Appendix Fig. S1 for sampling design; see Supplementary Table S1 for more details. Each 10 m × 10 m quadrat provided *a single sample*. This individual sample was the result of combining and mixing soil from 5 equal subsamples (each approximately 50 g) of soil from the top 10 cm, sampled using a small trowel marked to 10 cm depth starting down from the base of the litter layer. The five subsamples, one taken at each corner and one at the center, were gathered from each 0.01 ha area and mixed into a single soil sample bag^39^. Soil sample processing and DNA isolation were done one day after soil sampling. A list of the sampling sites and data on environmental parameters is provided as Supplementary Table S1. This study led to a total of 56 samples (7 sites × 4 biological replicate samples per site = 28 forest, and similarly 7 × 4 = 28 rubber plantation samples).

### Soil micro- and mesofaunal DNA extraction and PCR amplification

To maximize capture of soil microfauna and mesofauna, 200 g of each soil sample was suspended in around 2 I distilled water (DW) and then gently sieved through a set of sieves.

For the combined micro- & mesofauna isolation under the Baermann funnel method, we used three different sieves in a vertical series. We placed a 2 mm sieve at the top and a 150 µm sieve below it. Below this was a 20 µm sieve (coarse to fine from top to bottom). 2 I of soil suspension was slowly poured onto the 2 mm sieve. We gently washed down all of the soil particles that would pass through the 2 mm sieve using more distilled water, so that stones, roots, and litter, etc., remained on the 2 mm sieve. We then collected accumulated particles – containing fauna - from both the remaining sieves below (150 µm & 20 µm) using a wash bottle to wash through finer particles. Mesofauna would be selectively accumulated on top of the 150 µm sieve, and microfauna on top of the 20 µm sieve. The accumulated material from each sieve was removed by spatula and placed together onto cotton gauze in a Baermann funnel for further extraction followed by sugar flotation^40^.

As well as removing stones, roots, etc., the 2 mm sieve ensured that the macrofauna (>2 mm) did not end up in the collected material. Smaller eukaryotes (e.g., smaller protists) and clays, along with water, would be able to pass through the 20 µm holes of the finest sieve.

In this manner, we could collect both the micro- & mesofauna from the soil while keeping the contamination from macrofauna to a minimum. This collected soil was then used for both Baermann funnel and sugar flotation extraction. The Baermann Funnel method is most often used to capture Nematoda, but will also capture most other types of motile micro- & mesofaunal populations, as it depends on organismal locomotion^40,41^.

After 24–36 hours, fauna that had moved down out of the soil-derived material, to the water at the bottom of the funnel were collected by draining the fluid into a Falcon tube. Tubes were then centrifuged for 10 min approximately at 5000 rpm (settling micro- & mesofaunal populations at the bottom), and the individual sediment pellet was collected from each for DNA extraction^42^. Less active/dead components remaining in the soil material within the funnel were then captured by sugar flotation and centrifugation using a 40% (w/v) sugar solution, to yield a pellet in each tube^43^. This combined procedure was intended to increase the efficiency of capture of a range of micro- and mesofauna by capturing both active (funnel) and less active or dead components (flotation).

The extracted pellets from both methods were combined before DNA extraction at the tropical forest ecology lab in XTBG, China, using the MoBio Power Soil DNA isolation kit (MoBio Laboratories, Carlsbad, CA) according to the manufacturer’s instructions.

The isolated DNA was stored at −80 °C, and was later used as a template to amplify a ~400 bp diagnostic region, defined by primers NF1 (C. elegans 1226–1250 bp position) and 18Sr2b (C. elegans 1567–1588 bp position) towards the 3′ end of the 18S rDNA with PCR reaction conditions as described by Porazinska *et al*.^44^. Detailed PCR procedure is described in SI Appendix, Section 1b. This primer pair was originally designed for nematodes, but we were able to test its suitability in coverage for the whole of subkingdom Metazoa. We used the Ecotaxaspecificity module (http://pythonhosted.org/OBITools/scripts/ecotaxspecificity.html) under the ecoPrimers platform (http://pythonhosted.org/OBITools/scripts/ecoPrimers.html) which evaluates barcode resolution at different taxonomic ranks^45^. For the primer pair tested, ecoPrimers calculates a taxonomic coverage, which is the number of amplified target species relative to the total number of target species in the input database. Taxonomic coverage rates of 100% (SI Appendix: Section 1c,d) across all metazoan sequences for phyla in the SILVA SSU database suggest that the barcode designed for nematodes is degenerate enough to be used as a general primer for subkingdom Metazoa. All metazoan sequences across all phyla in the SILVA SSU database are predicted to amplify with this primer pair (Complete details can be found in SI Appendix: Section 1c,d).

### Data analysis

#### Sequence processing

Generated sequences were processed following Mothur’s 454 SOP^46^. All sequences shorter than 150 nucleotides, with homopolymers longer than eight nucleotides and all reads containing ambiguous base calls or incorrect primer sequences, were removed. Remaining sequences were aligned against the SILVA SSU eukaryotic aligned database. The sequences were further filtered to remove gaps generated after alignment, then preclustered using the Mothur implementation of pseudo-single linkage preclustering algorithm from Huse and colleagues^47^. Putative chimeric sequences were detected and removed via the Chimera Uchime algorithm contained within Mothur^48^ in *de novo* mode, which first splits sequences into groups and then checks each sequence within a group using the more abundant groups as a reference. Taxonomic classification of Metazoa was performed at a Bayesian cut-off of 55% with 1000 iterations against SILVA-ARB (database containing aligned 18S ribosomal RNA sequences with a minimum length of 1200 bases for Eukarya~ http://www.arb-silva.de/download/arb-files/; August 23, 2013). The remaining reads were then clustered using Mothur’s average algorithm. All samples were standardized by random subsampling to 1885 reads per sample (based on the smallest sample size by default), using the sub.sample command (http://www.mothur.org/wiki/Sub.sample), for calculating richness (OTUs at ≥99% sequence similarity), rarefaction curves, diversity and community compositional indices and matrices to be used later for the statistical analyses.

#### Statistical analyses

All data were analyzed using R 3.3.0^49^ except for NMDS analysis which was performed in Primer-6 Software. To test for differences in richness (OTUs), abundance (read counts), diversity indices (Shannon, and Faith Phylogenetic diversity; PD) and soil physicochemical parameters i.e., TOC (Total Organic Carbon), TN (Total Nitrogen), pH, SX (Soil Texture), ST (Soil Temperature), Ele (Elevation), GSW (Gravimetric Soil Water) & AP (Available Phosphorus), (SI Appendix: Section 1a) between forest and rubber plantation, we used GLMM with a Poisson distribution in package lme4 & nlme and site as a random term (for integer dataset: OTUs) and glmmPQL with a Quasi-Poisson distribution in package MASS as this allows quasi distributions with cluster as a blocking term (for non-integer data-set: abundance, Shannon, and PD etc.)^50^. The functions glmm and lmer do not work with a quasi-Poisson distribution^51^.

We employed the methods of Jost^52^ to multiplicatively partition γ-diversity into independent α- and β- components for each management type. We used the “multipart” function in Vegan package and ran 1000 simulations to compare values against a null model. Following Jost^52^, we partitioned species richness using a multiplicative framework, in which γ = α × β. We assessed α- & β- diversity at three spatial scales, such that overall γ- diversity within each forest was expressed as:

$${\rm{\gamma }}={\rm{\alpha }}.1\times {\rm{\alpha }}.2\times {\rm{\alpha }}.3\times {\rm{\beta }}.1\times {\rm{\beta }}.2\times {\rm{\beta }}.3$$

where α.1 & β.1 denote species richness and β- diversity between sites, respectively; α.2 & β.2 denote species richness and β- diversity between forest and rubber plantation, respectively; α.3 & β.3, denote species richness and β- diversity within sites, respectively. We then assessed whether forest clearance and conversion to plantation had influenced the β- diversity, by partitioning γ- diversity, separately for the forest samples and samples from rubber plantation, respectively.

We performed Non-metric Multi Dimensional Scaling (NMDS) to explore patterns in phylogenetic community composition using both the unweighted UniFrac & β-MNTD (β-mean nearest taxon distance) distance matrix with Primer-6 software^53^. The unweighted UniFrac measures the distance between two communities by calculating the fraction of the branch length in a phylogenetic tree that leads to descendants in either, but not both, of the two communities^54^. β-MNTD is the mean phylogenetic distance to the closest relative in a paired community for all taxa and is sensitive to the changes of lineages close to the phylogenetic tips^55^. We used an analysis of similarity (ANOSIM) with 999 permutations to test if community composition was significantly different between the forest and samples from rubber plantation. To test whether the taxonomic composition results may have been influenced by pseudoreplication or spatial arrangement of study sites (Ramage *et al*. 2013), we used a Mantel test under the function mantel (999 permutations/analysis) in package Vegan in R^56^ to compare the taxonomic composition to geographic distance between pairs of transects within a land-use type.

We performed a redundancy analysis (RDA) based variation partitioning analysis^57^ to assess the relative effects of environmental and spatial variables on community composition. We used Hellinger transformed OTU abundance data as the response variable and two sets of explanatory variables which included environmental variables (pH, Ele, TOC, AP, GSW, SX, TN, and ST) and spatial variables (geographical co-ordinates for sampling sites), respectively. Before the RDA, the environmental variables with high variance inflation factor (VIF) >10 were eliminated to avoid collinearity among factors. The importance of environmental and spatial variables in explaining species composition was determined by an RDA analysis using Monte Carlo permutation tests (999 unrestricted permutations) followed by forward selection to remove the non-significant variables from each of the explanatory sets.

To further evaluate the relative importance of each environmental variable and spatial distance on the community phylogenetic dissimilarity, we used a multiple regression on matrices (MRM) approach^58^. Before applying MRM to the dataset, we looked for redundant environmental factors using the VARCLUS procedure^59^ in the Hmisc R package^60^ and removed TN as it was highly correlated with TOC (Spearman’s ρ2 = 0.80). In MRM, non-significant factors were removed sequentially, and the analysis was repeated until only significant factors were left in the model. Significance was tested by permutations (9999), and P-values of the two-tailed tests are reported for this analysis.

#### Phylogenetic analysis

Phylogenetic analyses were performed to answer whether the community assembly in both the native forest and derived rubber plantations is influenced by deterministic or neutral processes (stochastic). A maximum-likelihood tree was constructed using aligned 18S rRNA gene sequences of representative OTUs in FastTree^61^. To evaluate the phylogenetic signal, an environmental optimum for each OTU was calculated for each environmental variable as in Stegen *et al*.^62^. Between-OTU environmental optimum differences were calculated as Euclidean distances using optima for all the environmental variables. The correlation coefficients between differences in environmental optima and phylogenetic distances were measured using Mantel correlogram with 999 random permutations^34,35^.

To calculate the turnover in phylogenetic community composition, we calculated the βMNTD^55,63^ using ‘comdistnt’ function (abundance.weighted = TRUE) of Picante R package^64^. Further, to investigate the influence of deterministic and stochastic processes on community assembly of soil micro- and mesofauna, a null modeling approach was implemented^34,35^. First, we calculated the β-nearest taxon index or βNTI, which is the difference between observed βMNTD and the null distribution (999 times) of βMNTD measured in units of its standard deviation. Values of βNTI < −2 and >+2 indicate significantly less and more than expected phylogenetic turnover (deterministic assembly), respectively. However, comparisons falling within the null distribution of βMNTD (|βNTI|<+2) indicate that the observed difference in community composition is not the result of deterministic selection and are instead attributable to stochastic assembly^63^.

## Results

The 18S rRNA gene metagenomic data (SI Appendix Fig. S2) gives details of both taxonomic identity and relative abundances of reads within each taxonomic category. It would be unrealistic to try to assign relative abundances of individuals to each taxonomic category since even closely related species of Metazoa from the same class or phylum can differ dramatically in size – and the samples pick up a great diversity of Metazoan taxa. For multicellular organisms, the number of cells and thus the number of copies of phylogenetic markers (such as 18S rRNA here) per individual are influenced by their size and their biomass. Consequently, under the premise that there was no technical bias, the number of reads obtained after PCR and NGS will probably be more closely correlated to their fractional biomass than abundance in terms of individuals.

### Land-use and community composition

The 56 soil samples from forest and rubber plantation yielded 4127 micro- and mesofaunal OTUs at ≥99% similarity from a total of 314,743 quality sequences. Out of the 4127 OTUs (without subsampling), 449 OTUs were shared between both land-use types (SI Appendix Fig. S3). Forest samples yielded 1864 unique OTUs and samples from rubber plantation had 1814 unique OTUs. At a subsampling size of 1885 reads, the mean number of OTUs per sample was 96 ± 31 (SD), ranging from 49 to 180 OTUs per sample.

In total, and for each of the two land-use types separately, the most abundant phylum was Annelida with 66.4% of all reads, followed by Arthropoda (15.5%), Nematoda (8.9%), Platyhelminthes (6.6%), Rotifera (1.3%), Tardigrada (0.25%) and Gastrotricha (0.16%). Of these, only annelids were significantly more abundant (as a proportion of reads) in rubber plantation than in forest (glmm PQL; t = 2.85, ρ < 0.05; SI Appendix Fig. S2).

**Figure 1:**
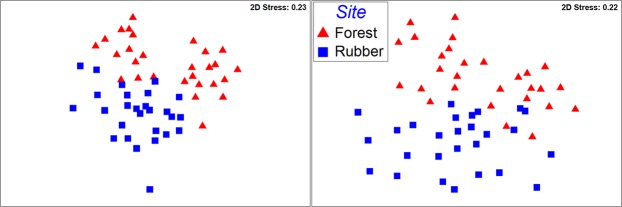
NMDS of metazoan community composition using the unweighted UniFrac (left); (2D stress: 0.23, 3D: stress 0.17) and β-MNTD index (right); (2D stress: 0.22, 3D: stress 0.16) in relation to land-use type.

The NMDS results revealed that phylogenetic community composition of both land-use types, based on both unweighted UniFrac and β-MNTD, was driven by forest conversion to rubber plantation, with samples from rubber plantation forming separate clusters apart from forest samples (Fig. 1; ANOSIM results, R = 0.3, ρ = 0.001). These results show clearly that the micro- and mesofaunal community composition of the rubber plantation differed from the original native forest. We did not find any significant effect of distance on taxonomic composition, by testing for phylogenetic signal using Mantel correlograms of soil microbiome within land-use types for forest samples (Mantel test, P > 0.05). However, we did find a significant effect of distance on composition matrices for samples from rubber plantation (Mantel test, R = 0.22, P < 0.05). As the correlation was weak, most of the variation observed in community composition may be explicable by land use change with a small fraction as a result of pseudoreplication. According to Oksanen^65^, true treatment replication is sometimes impossible to achieve in a study, and thus its absence need not weaken the value of a study, provided that the results are judged properly.

**Figure 2:**
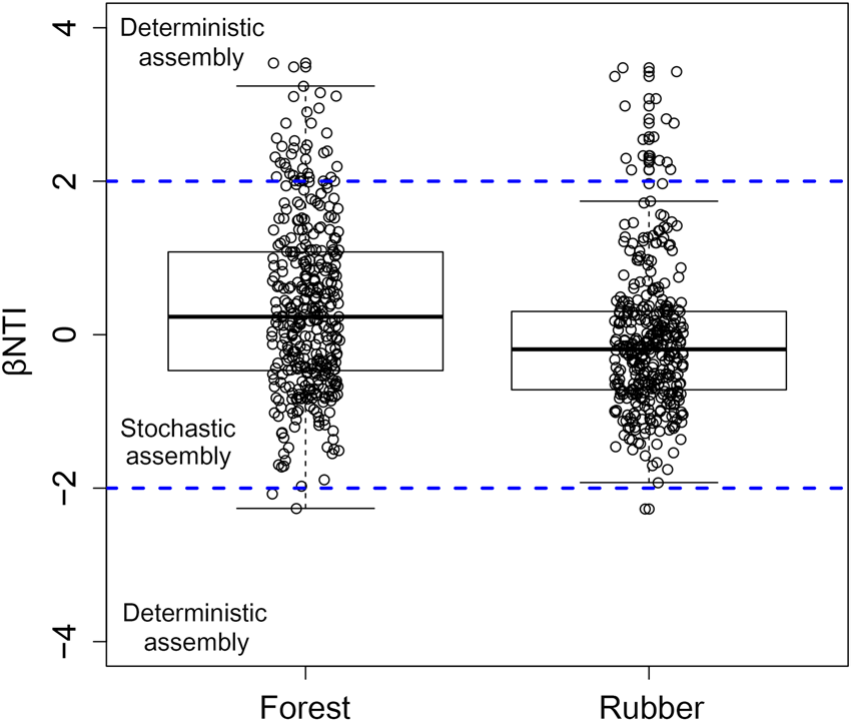
Patterns of βNTI across both land-use types. Horizontal dashed blue line indicate upper and lower significance thresholds at βNTI = +2 and −2, respectively.

Testing for phylogenetic signal using Mantel correlograms showed significant correlations between differences in OTU environmental optima and OTU phylogenetic distances but only across relatively short phylogenetic distances (ρ < 0.05, SI Appendix Fig. S4). We examined the relationship between βNTI and land-use type to infer changes in the relative influences of deterministic and stochastic assembly processes on the micro- and mesofaunal community. The pairwise comparisons of βNTI values within each land-use indicated similar patterns (Fig. 2). In forest and rubber plantation, the mean of pairwise βNTI values fell between the null distribution of βMNTD (|βNTI| <+2) indicating that community assembly is primarily influenced by stochastic processes.

### Land-use and diversity

**Figure 3:**
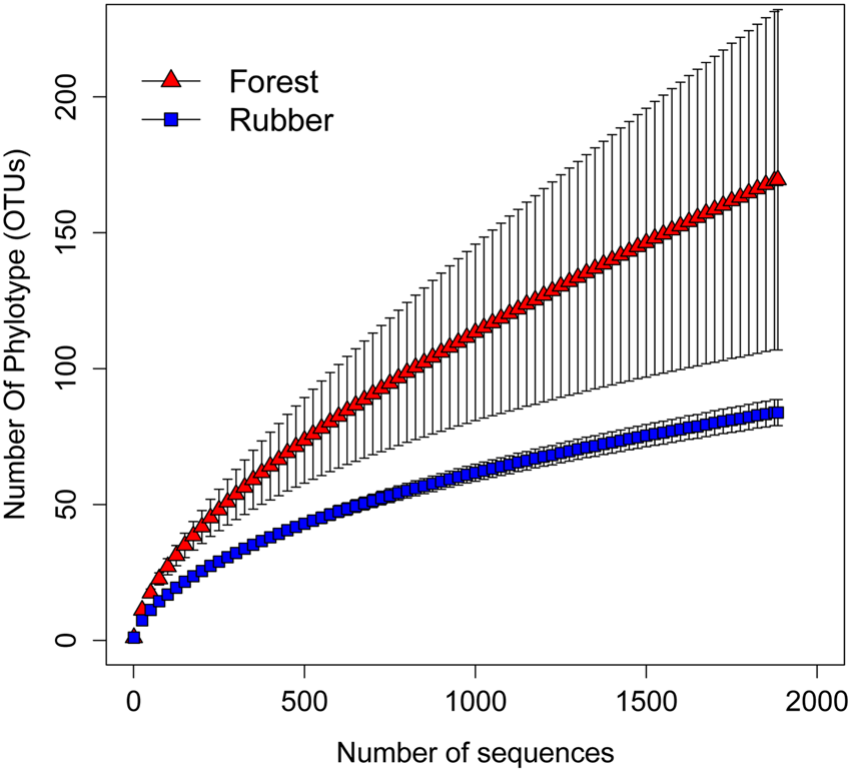
Rarefaction curve showing taxon richness of the operational taxonomic units (OTUs: ≥ 99% sequence similarity) of the 18S rRNA gene. OTUs average ± standard error (n = 28 for each forest and rubber plantation) at each rarefaction level is represented with triangles and squares respectively for the forest and samples from rubber plantation, respectively.

The rarefaction analysis showed that both richness and sample heterogeneity of OTUs were higher in the forest than in rubber plantation (Fig. 3). The α-diversity of 18S rRNA gene-based OTUs was significantly altered by forest conversion to rubber plantation. At a depth of 1885 reads, micro- and mesofaunal richness (OTUs) was significantly higher in the forest than in rubber plantation (GLMM; t = −2.28, p < 0.05). When measured separately for each of the three most abundant phyla (Table 1), only arthropods yielded significant results with OTU richness being higher in native forest (GLMM; t = −2.62, p < 0.01).

**Table 1:** Effect of forest conversion to rubber plantation on metazoa and three most dominant metazoan phyla, calculated by generalized linear mixed models. GLMM with a Poisson distribution (Richness) and site as a random term was used for integer dataset whereas glmm PQL with a Quasi-Poisson distribution (Diversity and Abundance) and site as a blocking term was used for non-integer dataset.

Taxa	Richness (OTUs)		Abundance (percent relative)		Diversity			
					Shannon		Faith’s PD	
	β ± SE	t/ρ	β ± SE	t/ρ	β ± SE	t/ρ	β ± SE	t/ρ
Metazoa	−0.25 ± 0.11	−2.28/0.02	—	—	−0.33 ± 0.07	−4.69/5e-04	−0.30 ± 0.08	−3.52/0.004
Annelida	—	—	0.29 ± 0.10	2.85/0.01	−0.30 ± 0.12	−2.51/0.03	—	—
Arthropoda	−0.26 ± 0.10	−2.62/0.009	—	—	—	—	−034 ± 0.11	−3.16/0.008
Nematoda	—	—	—	—	—	—	—	—

β ± SE stands for the coefficient ± standard error, t = t-value, ρ = ρ significance value. *Non-Significant values are not denoted in the table. A positive value for the coefficient (β) denotes that the tested property (Richness, Abundance or Diversity) is higher in rubber and vice-versa.

Other α-diversity indices also showed that diversity was significantly higher in native forests (glmm PQL results: Shannon; t = −4.7, p < 0.001, Faith’s PD; t = −3.5, p < 0.01), compared to the rubber plantations. Similar results were found when tested separately for each of the three major phyla present except for nematodes which did not yield any significant difference between the two land use types (Table 1).

Multiplicative diversity partitioning indicated that the native forest had significantly higher (p < 0.001) metazoan α- and β-diversity than rubber plantation at all levels in the hierarchy (Table 2). Multiplicative partitioning yielded similar results for analyses done separately for each of the three most abundant phyla (Table 2). Annelids, when taken separately, showed exactly the same patterns of diversity as observed for the total metazoan community. Nematodes and arthropods, however, showed a different pattern, with higher diversity in rubber plantation at some (but not all) hierarchal scales. For arthropods, α-diversity when measured between forest sites and for forest overall emerged as significantly higher than for rubber plantation. By contrast, beta diversity when calculated between rubber plantation sites, and for the total rubber plantation dataset, was significantly higher than for forest samples. For nematodes, at all levels in the taxonomic hierarchy, the diversity was higher in samples from rubber plantation - except for total beta diversity.

**Table 2:** Multiplicative diversity partitioning for the Metazoan community; γ-diversity was multiplicatively partitioned into independent α- and β-components following Jost^52^. (a) Separate runs for Native Forest and Rubber Plantation; (b) Single run for the overall dataset.

Group	Alpha.1	Alpha.2	Beta.1	Beta.2	Gamma		
**(a)**							
**Metazoa**							
Forest	14.28***	22.36***	2.79***	1.78***	39.86		
Rubber	6.79***	10.04***	2.29***	1.55***	15.55		
**Annelida**							
Forest	3.95***	5.48***	1.97***	1.42***	7.80		
Rubber	2.77***	3.51***	1.67***	1.32***	4.62		
**Arthropoda**							
Forest	7.15***	18.10***	6.95***	2.75***	49.69		
Rubber	5.39***	13.45***	7.49***	3.00***	40.38		
**Nematoda**							
Forest	13.42***	27.55***	5.63***	2.74***	75.56		
Rubber	14.26***	35.22***	6.67***	2.70***	95.14		
**(b)**							
**Group**	**Alpha.1**	**Alpha.2**	**Alpha.3**	**Beta.1**	**Beta.2**	**Beta.3**	**Gamma**
Metazoa	10.53***	16.2***	27.7***	2.78***	1.81***	1.07***	29.32
Annelida	3.36***	4.49***	6.20***	2.01	1.50	1.09	6.75
Arthropoda	6.27***	15.77***	45.04***	9.06	3.60	1.26	56.81
Nematoda	13.8***	31.39***	85.35***	8.56	3.77	1.39	118.41

*Alpha.1: between sites, Alpha.2: total, Beta1: between sites, Beta2: total.

*Alpha.1: between sites, Alpha.2: between forest and rubber plantation, Alpha.3: within sites, Beta1: between sites, Beta2: between forest and rubber plantation, Beta.3: within sites.

Significance level: ***p < 0.001, **p < 0.01, *p < 0.05.

**Figure 4:**
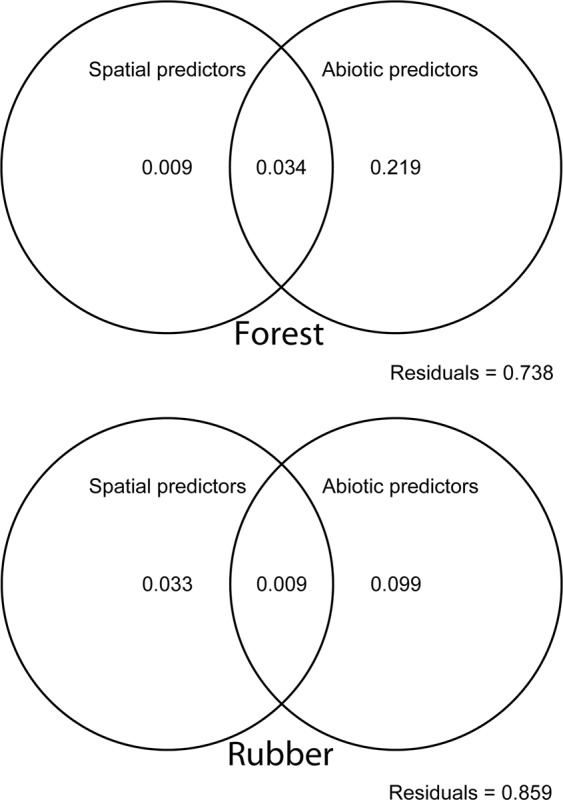
Variation partition analysis explaining the percentage of variation explained by abiotic (environmental distance) and spatial variables (geographical distance) of forest community and rubber monoculture community based on 18SrRNA gene sequences; see Materials & Methods section. Unique fractions of each explanatory set were evaluated by 999 permutations. Variation explained by abiotic variables were significant for both land-use types (forest: P < 0.005; rubber plantation: P < 0.01) whereas spatial variables were able to explain a significant part of variation for forest only (forest: P < 0.05).

Variation partitioning & RDA results revealed that in forest samples, environmental variables (i.e., pH, TOC & GSW) and Ele explained 25.3% of the total variation (ρ < 0.005) of metazoan communities, of which 21.9% of the total variation (ρ < 0.005) could be explained by soil variables alone (Fig. 4; top). In isolation, spatial variables were insignificant and could not explain any of the variation observed (ρ > 0.5). Likewise, within the rubber plantation metazoan community, environmental variables (pH & ST) and Ele explained the largest proportion (9.9%) of the total variation (ρ < 0.01) (Fig. 4; bottom). Unlike the forest, around 3.3% of total metazoan community variation in the rubber plantation could be explained by spatial variables (ρ < 0.05), while abiotic and spatial variables together explained around 14.1% of the variation (ρ < 0.01). Similar results were found for each of the three most abundant phyla (SI Appendix Fig. S5).

The MRM model explained a significant proportion of the variability in total phylogenetic community structure, with most of the environmental parameters in both land-use types (Table 3). Among the environmental variables measured, soil pH and TOC were correlated to UniFrac distance and β-MNTD for the total phylogenetic community for the forest whereas only soil pH was correlated to β-MNTD for the samples from rubber plantation. Spatial distance was significantly correlated to the unweighted UniFrac for both the forest and rubber plantation samples, but to β-MNTD only for samples from rubber plantation (Table 3).

**Table 3:** Multiple Regression on Matrices (MRM) analysis of metazoan communities using unweighted UniFrac and β-MNTD distance in forest and rubber plantation soils.

Measured parameters	UniFrac		β-MNTD	
	Forest	Rubber	Forest	Rubber
	R^2^ = 0.20***	R^2^ = 0.05***	R^2^ = 0.17***	R^2^ = 0.06***
pH	1.6 e-2***	NS	0.003***	1.571 e-6***
TOC	3.5 e-4***	NS	0.0002***	NS
Spatial Factors	5.3 e-8***	2.84 e-6***	NS	0.157***

The variation (R^2^) is explained by the remaining variables, and the partial regression coefficients of the final model are reported.

Significance level: ***p < 0.001, **p < 0.01, *p < 0.05, NS = non-significant.

When checked for differences between the two land-use types for major soil variables, a significant difference in TOC (glmm PQL; t = −2.8, p < 0.05) and a marginally significant difference in TN (glmm PQL; t = −2.0, p = 0.051), was present. Soil C: N ratio was also lower in samples from rubber plantation (p < 0.05).

## Discussion

### Impacts of conversion to plantation on community composition

Conversion of the forest to rubber plantation results in consistent changes in soil micro- & mesofaunal community composition. In comparison to rubber plantation, a larger proportion of variation for forest can be explained using abiotic parameters (Fig. 4, Variation Partitioning Results). Similarly, MRM results (Table 3) suggest that soil pH and TOC delimit the community composition to a certain extent, mostly for forest sites. It seems that for forest samples, environmental variables are a major force in structuring communities. There may be some analogy with a recent soil microfaunal study from Antarctica where microfaunal distribution was controlled by nutrients such as pH, organic matter and soil moisture^66^.

The phylogenetic signal^55,67^, detected across relatively short phylogenetic distances, indicates that closely related micro- and mesofaunal OTUs tend to occupy similar niches (ρ < 0.05, SI Appendix Fig. S4). Similar patterns have been observed in other studies of both prokaryotic and eukaryotic microbial communities^[62,68,69^. From phylogenetic null modeling^34^ we found a strong impact of stochastic processes on the assembly of micro- and mesofaunal communities in soils of both forest and rubber plantation, suggesting that despite its other evident effects, the shift to a highly altered rubber plantation environment has not altered the role of stochasticity in community structure.

According to other studies, conversion from forest to rubber plantation is also accompanied by a progressive increase in soil bulk density and continuous decrease in pore space (soil compaction) in the 0–10 cm soil layer^70,71^, which may decrease the niche availability for many small soil animals. There are also significant changes in the other soil properties^70,71^, which may affect the microbial community which is, in turn, grazed upon by many soil metazoans. Physical disturbance in rubber plantation sites at XTBG also results from the prevalent management practices, which might suppress metazoan abundances. Reports have shown positive effects of limiting physical disturbance on metazoan abundance, especially the nematode fauna^72^.

Reduction in soil carbon on conversion to rubber plantation may also be an important factor. Soil carbon provides a resource for fungi and bacteria, on which many soil micro- and mesofaunal community feeds, and thus changes in this food source could adversely affect their diversity. Also possibly important in producing lower soil micro- and mesofaunal diversity is the dominance of a non-native tree species (rubber) which fewer soil micro- and mesofauna may be adapted to feeding off.

Considering the phyla individually, annelids were significantly more abundant in the rubber plantation (p < 0.05, Table 1). We did not find any indication of invasive earthworm species such as *Pontoscolex corethrurus*^73^, and most reads belonged to highly diverse small annelid families (Enchytraeidae or potworms, Naididae & Tubificidae). Arthropoda, which comprises the vast majority of the described tropical rainforest fauna and is estimated in total at around 2.5–3.7 million species^74,75^ was represented by more OTUs in the forest (p < 0.01, Table 1). Soil calcium concentration is a limiting factor for terrestrial ostracods (Crustacea)^76^ and millipedes (Diplopoda)^77^.

Nematoda showed no significant changes in relative abundance with land-use change, although the family level feeding guild structure of the nematode community showed striking differences in relation to the land-use type (SI Appendix Fig. S6). An increase in Bacteria Feeding (BF) along with Fungi Feeding (FF) guilds in rubber plantation could be because the Omnivore Predator (OP) guild are sensitive to land use change and need more time to establish compared to more rapidly growing BF and FF nematodes^78,79^. The Plant-Feeding (PF) guild was significantly reduced in the rubber plantation soils, which is unsurprising as their abundance has been found to be directly correlated to plant community richness and soil C: N ratio^80^.

### Impacts of plantations on alpha-diversity

We predicted a substantial impact of forest conversion on soil micro- and mesofaunal α-diversity, and the results of this study suggest that indeed the conversion of the tropical forest of southern Yunnan to rubber plantation has resulted in a consistent decrease in α-diversity for soil micro- and mesofaunal community, except for nematodes, when these are considered separately. Possible reasons why α-diversity is lower in rubber plantations than in forest environments include: lack of small-scale heterogeneity in terms of various sizes/types of trees and shrubs, lower diversity of plants giving fewer organic substrates for niche differentiation in feeding, and a relative lack of carbon in litter and coarse debris flux resulting in fewer niches^81,82^.

Pesticides are generally used on rubber plantations, for example, to kill weeds, and are a possible factor producing the observed differences in soil metazoan diversity, as they are toxic to non-target species including soil animals^83,84^. A prevalent management practice in rubber plantations is repeated application of glyphosate, (alone or in a mixture with other pesticides)^31^ which increases the presence of glyphosate residues in soils^85–88^ and can be detected in top soils even after two years since the last spraying^86^. Glyphosate is reportedly a potent microbiocide^89,90^ and has been reported to severely affect the casting activities and reproduction of earthworms^91^.

Another conventional control practice used for powdery mildew and anthracnose diseases of rubber plantations is annual spraying of sulfur powder during the leaf expansion period of the rubber tree. This application over a period of time has been reported to reduce soil pH^92^, and it could be one of the reasons behind the lower pH observed in our samples from rubber plantation. However, in a study Li *et al*.^92^ sulfur spraying did not significantly alter the soil microbial community composition and function over the long term (48 years) some specific microorganisms, such as acidophilic bacteria, in the soil can use sulfur as an energy source and oxidize sulfur to SO_4_^2−^ accompanied with a decrease in soil pH which will persist over the years^93^. Changes in soil physicochemical characteristics and microbial community with the use of pesticides - as shown by these two examples - may eventually have effects on the micro- and mesofaunal populations of the rubber plantation soils.

Ecosystem engineers, such as earthworms, have been found to show a drastic decrease in abundance under rubber monoculture (possibly due to decline in soil exchangeable Ca^94^ and glyphosate herbicide^91^ in soil from rubber plantations). This may influence the diversity of other soil animals by regulating the availability, diversity, and spatial distribution of resources available to them^95,96^, and may help to explain the lower α-diversity in samples from rubber plantation.

### Impact of conversion to plantations on beta-diversity

As anticipated, and consistent with earlier studies of larger organisms, a decrease in micro- and mesofaunal β-diversity with the conversion from forest to rubber plantation was observed^97,98^ except for arthropods and nematodes. Still, total β-diversity for nematodes in samples from rubber plantation was marginally lower than forest samples (Table 2).

The uncoupling of α- and β-diversity after ecosystem conversion that is observed here for arthropods is generally not seen in examples from plants and larger animals^98^: conversion usually results in decreases in both α- and β-diversity in parallel as observed above for the total population. Although rarely found in higher organisms^99^ the converse situation – a decrease in β-diversity along with an increase in α-diversity in response to human disturbances has been reported elsewhere^100,101^. Similar uncoupling between α- and β-diversity has also been previously reported for bacterial communities, where the natural vegetation was either converted to agriculture^20^ or plantation^21^.

Various ecological mechanisms could be behind the greater arthropod β-diversity we observed in rubber plantations. With a random loss of the species present in the original rainforest (associated here with deforestation and initial and continuous human disturbance) and limited dispersal, species richness at the α-diversity level will be low and few species will be shared between disturbed plots. This will lead to an increased β-diversity between rubber plantation plots. Apart from the effects of dispersal limitation^102,103^, the increased arthropod β-diversity in rubber plantations might be a consequence of some unknown aspect of habitat heterogeneity that leads to species sorting by environmental selection^103,104^.

The diversity of a plant community is likely to be one of the major environmental factors that influences the diversity of the soil micro- and mesofaunal community, through shaping the resource availability to the soil community^105^. Although soil micro- and mesofauna need very small patch sizes and comparatively smaller sources of energy and nutrients to maintain their diversity, soil animal diversity is expected to increase with greater plant diversity due to enhanced opportunities for niche differentiation with respect to habitat use and sources of energy and nutrients^106–108^. However, the patterns are not consistent, and it has been often shown that the effect of plant species identity is greater than that of plant species richness^107,109–111^. The effect of plants on soil animal diversity is generally regulated in two different ways, i.e., availability of a resource and diversity of a resource^107,112^. Plants not only play a role in the availability of metabolic resources, but also have effects on other ecological factors that further shape soil habitats and niche space, including the presence and abundance of ecosystem engineers^95,96^, microclimatic conditions, and general soil properties.

## Conclusion

It is clear that conversion of forest to rubber plantation involves a major turnover of lower level taxa (expressed as OTUs) of micro- and mesofauna, and an overall loss of diversity. Hypothetically, this loss of micro- and mesofaunal diversity could result in lower soil and ecosystem resilience, by analogy with other systems where removal of diversity has resulted in decreased stability^112^. It is also relevant to consider whether the loss of the original rainforest soil micro- and mesofauna might make it more difficult to re-establish tropical rainforest on former rubber plantations, as these organisms have important and specialized roles in nutrient cycling in tropical forests. Such questions should be investigated in further studies of this and other analogous systems.

## Supplementary information

Supplementary_Appendix S1

Supplementary_Table_S1

## Data Availability

DNA sequences: All data are available from Figshare (Metadata: 10.6084/m9.figshare.3506822.v2; Sequence data: 10.6084/m9.figshare.7842029). Datasets and metadata are published under the CCo license. R scripts: available upon request; please email singhdharmesh24@gmail.com for R scripts derived from freely available R packages online: see material and methods for more details. Site information such sample locations with coordinates, vegetation types and other physiochemical properties of the soils used in this study: supplied as a supporting document with the manuscript; see Supplementary Table 1 for more details.

## References

[1] Dirzo R, Raven PH (2003). Global state of biodiversity and loss. Annu. Rev. Env. Resour..

[2] Wright SJ (2005). Tropical forests in a changing environment. Trends Ecol. Evol..

[3] Gibson L (2011). Primary forests are irreplaceable for sustaining tropical biodiversity. Nature.

[4] Gibbs HK (2010). Tropical forests were the primary sources of new agricultural land in the 1980s and 1990s. P. Natl. Acad. Sci. USA.

[5] Sodhi NS, Koh LP, Brook BW, Ng PKL (2004). Southeast Asian biodiversity: an impending disaster. Trends Ecol. Evol..

[6] FAO. Forests for improved nutrition and food security. Rome. www.fao.org/forestry/27976-02c09ef000fa99932eefa37c22f76a055.pdf (2010).

[7] Li Z, Fox JM (2012). Mapping rubber tree growth in mainland Southeast Asia using time-series MODIS 250 m NDVI and statistical data. Appl. Geogr..

[8] Danielsen, F. & Heegaard, M. In *Management of Tropical Forests: Towards an Integrated Perspective* (ed. Øyvind, Sandbukt) 73–92 (Centre for Development and the Environment, University of Oslo, 1995).

[9] Aratrakorn S, Thunhikorn S, Donald PF (2006). Changes in bird communities following conversion of lowland forest to oil palm and rubber plantations in southern Thailand. Bird Conserv. Int..

[10] Wu Z-L, Liu H-M, Liu L-Y (2001). Rubber cultivation and sustainable development in Xishuangbanna, China. Int. J. Sust. Dev. World.

[11] Zhang M, Fu XH, Feng WT, Zou X (2007). Soil organic carbon in pure rubber and tea-rubber plantations in South-western China. Trop. Ecol..

[12] Ziegler AD (2012). Carbon outcomes of major land-cover transitions in SE Asia: great uncertainties and REDD plus policy implications. Global Change Biol..

[13] Li HM, Ma YX, Liu WJ, Liu WJ (2009). Clearance and fragmentation of tropical rain forest in Xishuangbanna, SW, China. Biodivers. Conserv..

[14] Guardiola-Claramonte M (2008). Local hydrologic effects of introducing non-native vegetation in a tropical catchment. Ecohydrology.

[15] Tan, Z. H. *et al*. Rubber plantations act as water pumps in tropical China. *Geophys. Res. Lett*. **38** (2011).

[16] Xu JC, Grumbine RE, Beckschafer P (2014). Landscape transformation through the use of ecological and socioeconomic indicators in Xishuangbanna, Southwest China, Mekong Region. Ecol. Indic..

[17] McGrath DA, Smith CK, Gholz HL, Oliveira FD (2001). Effects of land-use change on soil nutrient dynamics in Amazonia. Ecosystems.

[18] Murty, D., Kirschbaum, M. U. F., McMurtrie, R. E. & McGilvray, A. Does conversion of forest to agricultural land change soil carbon and nitrogen? A review of the literature. *Global Change Biol*. **8** (2002).

[19] Stocking MA (2003). Tropical soils and food security: The next 50 years. Science.

[20] Rodrigues JLM (2013). Conversion of the Amazon rainforest to agriculture results in biotic homogenization of soil bacterial communities. P. Natl. Acad. Sci. USA.

[21] Lee-Cruz L, Edwards DP, Tripathi BM, Adams JM (2013). Impact of Logging and Forest Conversion to Oil Palm Plantations on Soil Bacterial Communities in Borneo. Appl. Environ. Microb..

[22] Lan GY (2017). Change in Soil Microbial Community Compositions and Diversity Following the Conversion of Tropical Forest to Rubber Plantations in Xishuangbanna, Southwest China. Trop. Conserv. Sci..

[23] Barrios E (2007). Soil biota, ecosystem services and land productivity. Ecol. Econ..

[24] Danovaro R (2008). Exponential decline of deep-sea ecosystem functioning linked to benthic biodiversity loss. Curr. Biol..

[25] Wardle DA (2006). The influence of biotic interactions on soil biodiversity. Ecol. Lett..

[26] Neher DA (2001). Role of nematodes in soil health and their use as indicators. J. Nematol..

[27] Behnke A (2006). Microeukaryote community patterns along an O-2/H2S gradient in a supersulfidic anoxic Fjord (Framvaren, Norway). Appl. Environ. Microb..

[28] Richards TA, Bass D (2005). Molecular screening of free-living microbial eukaryotes: diversity and distribution using a meta-analysis. Curr. Opin. Microbiol..

[29] Groisillier A, Massana R, Valentin K, Vaulotl D, Guilloul L (2006). Genetic diversity and habitats of two enigmatic marine alveolate lineages. Aquat. Microb. Ecol..

[30] Creer S (2010). Ultrasequencing of the meiofaunal biosphere: practice, pitfalls and promises. Mol. Ecol..

[31] Xiao HF (2014). Intensive rubber cultivation degrades soil nematode communities in Xishuangbanna, southwest China. Soil Biol. & Biochem..

[32] Bik HM (2012). Sequencing our way towards understanding global eukaryotic biodiversity. Trends Ecol. Evol..

[33] Ofiteru ID (2010). Combined niche and neutral effects in a microbial wastewater treatment community. P. Natl. Acad. Sci. USA.

[34] Stegen JC (2013). Quantifying community assembly processes and identifying features that impose them. Isme J..

[35] Wang JJ (2013). Phylogenetic beta diversity in bacterial assemblages across ecosystems: deterministic versus stochastic processes. Isme J..

[36] Li YW, Deng XB, Cao M, Lei YB, Xia YJ (2013). Soil restoration potential with corridor replanting engineering in the monoculture rubber plantations of Southwest China. Ecol. Eng..

[37] Zhu H (1992). The tropical rainforest vegetation in Xishuangbanna. Chin. Geogr. Sci.

[38] Zhu H, Cao M, Hu HB (2006). Geological history, flora, and vegetation of Xishuangbanna, southern Yunnan, China. Biotropica.

[39] Fulthorpe RR, Roesch LFW, Riva A, Triplett EW (2008). Distantly sampled soils carry few species in common. Isme J..

[40] Baermann G (1917). Eine einfache Methode zur Auffindung von Ancyclostomum (Nematode) Larven in Erdproben. Nederlands Tijdscrift Voor Geneeskunde..

[41] Whitehead AG, Hemming JR (1965). A Comparison of Some Quantitative Methods of Extracting Small Vermiform Nematodes from Soil. Ann. Appl. Biol..

[42] Viglierchio DR, Schmitt RV (1983). On the Methodology of Nematode Extraction from Field Samples - Baermann Funnel Modifications. J. Nematol..

[43] Jenkins WR (1964). A rapid centrifugal-flotation technique for separating nematodes from soil. Plant Dis. Rep..

[44] Porazinska DL (2009). Evaluating high-throughput sequencing as a method for metagenomic analysis of nematode diversity. Mol. Ecol. Resour..

[45] Riaz T (2011). EcoPrimers: inference of new DNA barcode markers from whole genome sequence analysis. Nucleic Acids. Res..

[46] Schloss PD, Gevers D, Westcott SL (2011). Reducing the Effects of PCR Amplification and Sequencing Artifacts on 16S rRNA-Based Studies. Plos One.

[47] Huse SM, Welch DM, Morrison HG, Sogin ML (2010). Ironing out the wrinkles in the rare biosphere through improved OTU clustering. Environ. Microbiol..

[48] Edgar RC, Haas BJ, Clemente JC, Quince C, Knight R (2011). UCHIME improves sensitivity and speed of chimera detection. Bioinformatics.

[49] R: A language and environment for statistical computing (R Foundation for Statistical Computing, Vienna, Austria, 2016).

[50] Zuur, A. F., Ieno, E. N., Walker, N. J., Saveliev, A. A. & Smith, G. M. *Mixed effects models and extensions in ecology with R*. (Springer, New York, 2009).

[51] Bates D, Machler M, Bolker BM, Walker SC (2015). Fitting Linear Mixed-Effects Models Using lme4. J. Stat. Softw..

[52] Jost L (2007). Partitioning diversity into independent alpha and beta components. Ecology.

[53] Clarke, K. R. & Gorley, R. N. *Primer v6: User Manual/Tutorials* (Primer-E Ltd.: Plymouth, UK., 2006).

[54] Lozupone C, Knight R (2005). UniFrac: a new phylogenetic method for comparing microbial communities. Appl. Environ. Microb..

[55] Fine PV, Kembel SW (2011). Phylogenetic community structure and phylogenetic turnover across space and edaphic gradients in western Amazonian tree communities. Ecography.

[56] Oksanen J (2013). Package ‘vegan’. R Packag ver.

[57] Peres-Neto PR, Legendre P, Dray S, Borcard D (2006). Variation partitioning of species data matrices: Estimation and comparison of fractions. Ecology.

[58] Legendre P, Lapointe FJ, Casgrain P (1994). Modeling brain evolution from behavior: A permutational regression approach. Evolution.

[59] Sarle, W. S. *The VARCLUS Procedure. SAS/STAT User’s Guide*. 4th edn, (Cary NC: SAS Institute, Inc., 1990).

[60] Harrell, F. E. & Dupont, C. Hmisc: Harrell miscellaneous. R package version 3.9-3. Available at http://CRAN.R-project.org/package=Hmisc (2012).

[61] Price MN, Dehal PS, Arkin AP (2010). FastTree 2-Approximately Maximum-Likelihood Trees for Large Alignments. Plos One.

[62] Stegen JC, Lin X, Konopka AE, Fredrickson JK (2012). Stochastic and deterministic assembly processes in subsurface microbial communities. Isme J..

[63] Kembel SW (2010). Picante: R tools for integrating phylogenies and ecology. Bioinformatics.

[64] Oksanen L (2004). The devil lies in details: reply to Stuart Hurlbert. Oikos.

[65] Smykla J (2018). Geochemical and biotic factors influencing the diversity and distribution of soil microfauna across ice-free coastal habitats in Victoria Land, Antarctica. Soil Biol. & Biochem..

[66] Cavender-Bares J, Kozak KH, Fine PVA, Kembel SW (2009). The merging of community ecology and phylogenetic biology. Ecol. Lett..

[67] Moroenyane I, Chimphango SBM, Wang J, Kim HK, Adams JM (2016). Deterministic assembly processes govern bacterial community structure in the Fynbos, South Africa. Microb. Ecol..

[68] Tripathi BM (2018). Soil pH mediates the balance between stochastic and deterministic assembly of bacteria. Isme J..

[69] Dawoe EK, Quashie-Sam JS, Oppong SK (2014). Effect of land-use conversion from forest to cocoa agroforest on soil characteristics and quality of a Ferric Lixisol in lowland humid Ghana. Agroforest. Syst..

[70] Li HM, Ma YX, Liu WJ, Liu WJ (2012). Soil Changes Induced by Rubber and Tea Plantation Establishment: Comparison with Tropical Rain Forest Soil in Xishuangbanna, SW China. Environ Manage.

[71] Nakamoto, T., Yamagishi, J. & Miura, E. Effect of reduced tillage on weeds and soil organisms in winter wheat and summer maize cropping on Humic Andosols in Central Japan. *Soil Till. Res*. **85** (2006).

[72] Gonzalez G, Huang CY, Zou XM, Rodriguez C (2006). Earthworm invasions in the tropics. Biol. Invasions.

[73] Hamilton AJ (2010). Quantifying Uncertainty in Estimation of Tropical Arthropod Species Richness. Am. Nat..

[74] Basset Y (2012). Arthropod Diversity in a Tropical Forest. Science.

[75] Ohta T, Niwa S, Agetsuma N, Hiura T (2014). Calcium concentration in leaf litter alters the community composition of soil invertebrates in warm-temperate forests. Pedobiologia.

[76] Golovatch SI, Kime RD (2009). Millipede (Diplopoda) distributions: A review. Soil Organisms.

[77] Ferris H, Bongers T, de Goede RGM (2001). A framework for soil food web diagnostics: extension of the nematode faunal analysis concept. Appl. Soil. Ecol..

[78] Liang WJ (2009). Nematode faunal response to long-term application of nitrogen fertilizer and organic manure in Northeast China. Soil Biol. & Biochem..

[79] Cortois R (2017). Possible mechanisms underlying abundance and diversity responses of nematode communities to plant diversity. Ecosphere.

[80] Zak DR, Holmes WE, White DC, Peacock AD, Tilman D (2003). Plant diversity, soil microbial communities, and ecosystem function: Are there any links. Ecology.

[81] Bezemer TM (2010). Divergent composition but similar function of soil food webs of individual plants: plant species and community effects. Ecology.

[82] Relyea RA (2005). The impact of insecticides and herbicides on the biodiversity and productivity of aquatic communities. Ecol. Appl..

[83] Rahman PM, Varma RV, Sileshi GW (2012). Abundance and diversity of soil invertebrates in annual crops, agroforestry and forest ecosystems in the Nilgiri biosphere reserve of Western Ghats, India. Agroforest. Syst..

[84] Al-Rajab AJ, Amellal S, Schiavon M (2008). Sorption and leaching of (14)C-glyphosate in agricultural soils. Agron. Sustain. Dev..

[85] Bergstrom L, Borjesson E, Stenstrom J (2011). Laboratory and Lysimeter Studies of Glyphosate and Aminomethylphosphonic Acid in a Sand and a Clay Soil. J. Environ. Qual..

[86] Borggaard OK, Gimsing AL (2008). Fate of glyphosate in soil and the possibility of leaching to ground and surface waters: a review. Pest. Manag. Sci..

[87] Simonsen L, Fomsgaard IS, Svensmark B, Spliid NH (2008). Fate and availability of glyphosate and AMPA in agricultural soil. J. Environ. Sci. Heal. B..

[88] Zobiole LHS, Kremer RJ, Oliveira RS, Constantin J (2011). Glyphosate affects micro-organisms in rhizospheres of glyphosate-resistant soybeans. J. Appl. Microbiol..

[89] Druille M, Cabello MN, Omacini M, Golluscio RA (2013). Glyphosate reduces spore viability and root colonization of arbuscular mycorrhizal fungi. Appl. Soil. Ecol..

[90] Gaupp-Berghausen M, Hofer M, Rewald B, Zaller JG (2015). Glyphosate-based herbicides reduce the activity and reproduction of earthworms and lead to increased soil nutrient concentrations. Sci. Rep.-UK.

[91] Li YW (2016). Accumulated Impacts of Sulfur Spraying on Soil Nutrient Availability and Microbial Biomass in Rubber Plantations. Clean-Soil Air Water.

[92] Wang YP (2008). Effect of sulphur on soil Cu/Zn availability and microbial community composition. J. Hazard. Mater..

[93] Aweto AO (1987). Physical and Nutrient Status of Soils under Rubber (Hevea-Brasiliensis) of Different Ages in Southwestern Nigeria. Agr. Syst..

[94] Lavelle P (1997). Soil function in a changing world: the role of invertebrate ecosystem engineers. Eur. J. Soil Biol..

[95] Eisenhauer N (2010). The action of an animal ecosystem engineer: Identification of the main mechanisms of earthworm impacts on soil microarthropods. Pedobiologia.

[96] Philpott SM (2008). Biodiversity Loss in Latin American Coffee Landscapes: Review of the Evidence on Ants, Birds, and Trees. Conserv. Biol..

[97] Bierregaard, R. O. Jr., Gascon, C., Lovejoy, T. E. & Mesquita, R. C. G. 496 (Yale University Press, New Haven & London, 2001).

[98] Olden JD, Poff NL (2003). Toward a mechanistic understanding and prediction of biotic homogenization. Am. Nat..

[99] Hewitt J, Thrush S, Lohrer A, Townsend M (2010). A latent threat to biodiversity: consequences of small-scale heterogeneity loss. Biodivers. Conserv..

[100] Smart SM (2006). Biotic homogenization and changes in species diversity across human-modified ecosystems. P. R. Soc. B..

[101] Chase JM, Myers JA (2011). Disentangling the importance of ecological niches from stochastic processes across scales. Philos. T. R. Soc. B..

[102] Hanson CA, Fuhrman JA, Horner-Devine MC, Martiny JBH (2012). Beyond biogeographic patterns: processes shaping the microbial landscape. Nat. Rev. Microbiol..

[103] Kallimanis AS (2008). How does habitat diversity affect the species-area relationship?. Global Ecol. Biogeogr..

[104] Sylvain ZA, Wall DH (2011). Linking Soil Biodiversity and Vegetation: Implications for a Changing Planet. Am. J. Bot..

[105] Anderson JM (1978). Inter-Habitat and Intra-Habitat Relationships between Woodland Cryptostigmata Species-Diversity and Diversity of Soil and Litter Microhabitats. Oecologia.

[106] Wardle DA, Yeates GW, Barker GM, Bonner KI (2006). The influence of plant litter diversity on decomposer abundance and diversity. Soil Biol. & Biochem..

[107] Coleman DC (2008). From peds to paradoxes: Linkages between soil biota and their influences on ecological processes. Soil Biol. & Biochem..

[108] Wardle DA, Yeates GW, Williamson W, Bonner KI (2003). The response of a three trophic level soil food web to the identity and diversity of plant species and functional groups. Oikos.

[109] De Deyn GB, Raaijmakers CE, van Ruijven J, Berendse F, van der Putten WH (2004). Plant species identity and diversity effects on different trophic levels of nematodes in the soil food web. Oikos.

[110] Eissfeller V, Langenbruch C, Jacob A, Maraun M, Scheu S (2013). Tree identity surpasses tree diversity in affecting the community structure of oribatid mites (Oribatida) of deciduous temperate forests. Soil Biol. & Biochem..

[111] Hooper DU (2000). Interactions between aboveground and belowground biodiversity in terrestrial ecosystems: Patterns, mechanisms, and feedbacks. Bioscience.

[112] Strecker T, Mace OG, Scheu S, Eisenhauer N (2016). Functional composition of plant communities determines the spatial and temporal stability of soil microbial properties in a long-term plant diversity experiment. Oikos.

