# Supplementary material for: Tropical forest conversion to rubber plantation affects soil micro- & mesofaunal community & diversity
Source: Sci Rep. 2019 Apr 10;9:5893. doi: 10.1038/s41598-019-42333-4 (PMC6458137; doi:10.1038/s41598-019-42333-4)
Supplement: Supplementary file 1 — Supplementary_Appendix S1 [file 41598_2019_42333_MOESM1_ESM.docx]

Supplementary Information Appendix S1 For:

Tropical forest conversion to rubber plantation affects soil micro- & mesofaunal community & diversity

Dharmesh Singh^1, 2^, J. W. Ferry Slik^3^, Yoon-Seong Jeon^4^, Kyle W. Tomlinson^1^, Xiaodong Yang^5^, Jin Wang^5^, Dorsaf Kerfahi^6^, Dorota L Porazinska^7^, and Jonathan M. Adams^8^*

This file includes:

SI Materials and Methods

Fig. S1

Fig. S2

Fig. S3

Fig. S4

Fig. S5

Fig. S6

1. **Materials and Methods**
   1. **Field Sampling**

Sampling was carried out during the monsoon wet season month of August 2013. Seven rainforest sampling sites were chosen within a 10 km radius of XTBG. Rubber plantation plots were chosen adjacent to each old-growth forest sampling site. At each sampling site, four quadrats (10 m x 10 m in size), located at least 30 m apart along a linear transect were collected leading to a total of 56 samples (7x4=28 forest, 7x4=28 rubber plantation); see Fig. S1 for sampling design; see Supplementary Table S1 for more details. Each individual sample consisted of 5 equal subsamples (approximately 50 g) of soil from the top 10 cm, sampled using a small trowel marked to 10 cm depth underneath the litter layer. The five subsamples, one taken at each corner and one at the center, were gathered from each 0.01 ha area, and mixed into a single soil sample bag. A list of the sampling sites and data on environmental parameters is provided as Supplementary Table S1. Soil temperature (ST) was measured by burying soil thermometers at a depth of 10 cm for 10 minutes or until the reading became constant. Since 10 cm depth usually averages temperatures over several weeks ^1^, this is a more accurate representation of general soil temperature than a near-surface measurement. Elevation (Ele), geographical co-ordinates (both measured using a GPS device) and ST were measured at the site itself, while other soil parameters such as total nitrogen (TN), total organic carbon (TOC), pH, available phosphorus (AP), gravimetric soil water (GSW) and soil texture (SX) were estimated at the Biogeochemical laboratory, XTBG, Xishuangbanna using standard methods; Supplementary Table S1.

- 1. **PCR Amplification**

The isolated DNA was stored at - 80°C, and was later used as a template to amplify a ~400 bp diagnostic region, defined by primers NF1 (C. elegans 1226-1250 bp position) and 18Sr2b (C. elegans 1567-1588 bp position) towards the 3’ end of the 18S rDNA with PCR reaction conditions as described by Porazinska et al ^2^. PCR amplification was performed in 50 μl reactions (triplicates) with reaction mixture composed of 1 μl of DNA extract, 0.4μM of each primer, 0.2 mM of each dNTP mix, 10X Taq Reaction Buﬀer (25mM MgCl_2_ pre-mixed) and 1.25 U SolgTM Taq DNA Polymerase (SolGent co., Ltd., Korea) using the following program: 95°C for 2 min; 30 cycles of 95°C for 1 min, 50°C for 45 s, 72°C for 3 min; and 72°C for 10 min. Amplified products (triplicates; visualized separately on 1% agarose gel) for every sample were collected into a single vial before purification. Negative control (no-template) was included in all the PCR reactions to check for contamination. Purified (using QIAquick PCR purification kit, Qiagen as per the manufacturer’s instructions) amplified product was pyrosequenced using a 454 GS-FLX Titanium system (Roche). Control samples showed no presence of genomic DNA when visualized by electrophoresis in 1% agarose gel.)

- 1. **Ecotaxaspecificity run using Ecoprimers software**

# **Objective**

To check whether the primer pair NF1 (*C. elegans* 1226-1250 bp position) and 18Sr2b (*C. elegans* 1567-1588 bp position) ^2^ earlier designed for the phylum Nematoda (taxon under sub-kingdom Metazoa) can be used as such for amplifying Metazoa in general. This is because our results from NGS sequencing contained sequences from across several different phyla in the sub-kingdom Metazoa (including Nematoda at around 10% of reads).

# **Methodology**

To check primer range, we used the Ecotaxaspecificity module (<http://pythonhosted.org/OBITools/scripts/ecotaxspecificity.html>) under the Ecoprimers platform (<http://pythonhosted.org/OBITools/scripts/ecoPrimers.html>) which in simple terms, evaluates barcode resolution at different taxonomic ranks ^3^. In our study, we were most interested in studying the range of the primer at sub-Kingdom level (Metazoa).

As inputs, Ecotaxaspecificity takes a sequence record file annotated with taxids (taxonomic IDs) in the sequence header, and a database formatted as an ecopcr database. This ecopcr database can be generated using the obitaxonomy command ([http://pythonhosted.org/OBITools/scripts/obitaxonomy.html#module-obitaxonomy](http://pythonhosted.org/OBITools/scripts/obitaxonomy.html)) in the ecoprimers platform.

## Input:

a. Metazoan Database file composed of sequences extracted from SILVA SSU database file and then converted using obiconvert into ecoPCR db file. (The number of sequences in the database for Metazoa was 12383 and for Nematoda was 1120)

b. Primer set used (removing all adaptor, barcode and linker sequences)



## Program output:

## Input primer sequences for ecotaxspecificity, which are saved in unique.primer.fasta

>PR1 taxid=6231;

GGTGGTGCATGGCCGTTCTTAGTT

>PR2 taxid=6231;

CCTACAAAGGGCAGGGACGTAAT

[root@blackwidow marketing]# tool/OBITools-1.2.0/OBITools-1.2.0/bin/ecotaxspecificity -d my_db/mtza unique.primer.fasta

Reading binary taxonomy database...

[INFO : Taxon alias file found]

[INFO : Local taxon file not found]

Taxonomical tree read

[INFO : Preferred taxon name file not found]

ok

unique.primer.fasta 100.0 % |##################################################\] remain : 00:00:00

Alignment : 1 x 2 -> 0 50.0 % |#########################\ ] remain : 00:00:00

rank taxon_ok taxon_total percent

superkingdom 1 1 100.00

kingdom 1 1 100.00

phylum 1 1 100.00

# **Results**

“taxon_total” refers to the percentage of different taxa observed at this rank in the sequence record file (when taxonomic information is available at this rank), and “taxon_ok” corresponds to the number of taxa that the barcode sequence identifies unambiguously in the taxonomic database. In our case, since the sequence records or database contains only sequences from a single sub-kingdom Metazoa, taxon total corresponds to 1, and taxon ok also corresponds to a single kingdom. “percent” is the percentage of unambiguously identified taxa among the total number of taxa (taxon_ok/taxon_total*100). A 100 percent here suggests that the barcode here designed for Nematode is degenerate enough to be used as such as a general primer for sub-kingdom Metazoa. All metazoan sequences across all phyla in the SILVA SSU database are predicted to amplify with this primer pair.

# **Conclusions**

Since all Metazoa sequences across all phyla in the SILVA SSU database are predicted to amplify with this primer, this primer can be regarded as a suitable universal primer for Metazoa.

# **Primer Specificity Test**

To estimate the primer specificity, in-house sequence alignment program was used implementing pairwise sequence alignment algorithm^4^. Both forward NF1 (C. elegans 1226-1250 bp position) and reverse 18Sr2b (C. elegans 1567-1588 bp position) primer sequences ^2^, and SILVA SSU database sequence file were used as an input of this program. A sequence in the database was considered to be virtually amplified when both primer sequences were aligned to the sequence with ≥90% similarity.

# **Results**

Alignment tests showed that around 90.3% (11176 out of total 12383) of the metazoan sequences could be aligned successfully against the primer pair used, at an alignment cut off of ≥90% similarity which in other words suggests that >90% of the metazoan sequences across all phyla in the SILVA SSU database are predicted to be amplified using this primer pair.

# **Conclusions**

Since >90% metazoan sequences across all phyla in the SILVA SSU database are predicted to amplify with this primer, this primer can be regarded as a suitable universal primer for Metazoa.

**SI Figure Captions**

Fig. S1: Sampling scheme with the four sampling points within a site (located 30m apart). Each individual sample consisted of 5 equal subsamples (red dots within the enlarged view of a single individual sample), one taken at each corner and one at the center, were gathered from each 0.01 ha area, and mixed.

Fig. S2: Percent relative abundance of total metazoan reads (top) and the most abundant metazoan phyla among forest and rubber samples (bottom). Abbreviations: RF, forest; RP, rubber.

Fig. S3: Venn diagram representing the shared and unique OTUs between the land-use types. Quality reads clustered at a ≥99% pairwise identity threshold were used to generate the diagram using Venn command (MiSeq SOP; ^5^).

Fig. S4: Mantel correlogram between the pairwise matrix of OTU environmental optima and phylogenetic distances. Closed squares represent significant phylogenetic signals at the significance level of α<0.05 after Bonferroni correction for multiple testing.

Fig. S5: Percentage of variation explained by abiotic (environmental distance) and spatial variables (geographical distance) of forest community and rubber monoculture community based on 18SrRNA gene sequences. Unique fractions of each explanatory set were evaluated by 999 permutations.

Fig. S6: Relative abundance (%) of nematode feeding guilds at the family level. PF: Plant Feeding; FF: Fungi Feeding; BF: Bacteria Feeding; OP: Omnivore/Predator. Note the sum of the relative abundance of the four feeding groups is less than 100 percent, due to a sizeable proportion of unclassified sequences.

#
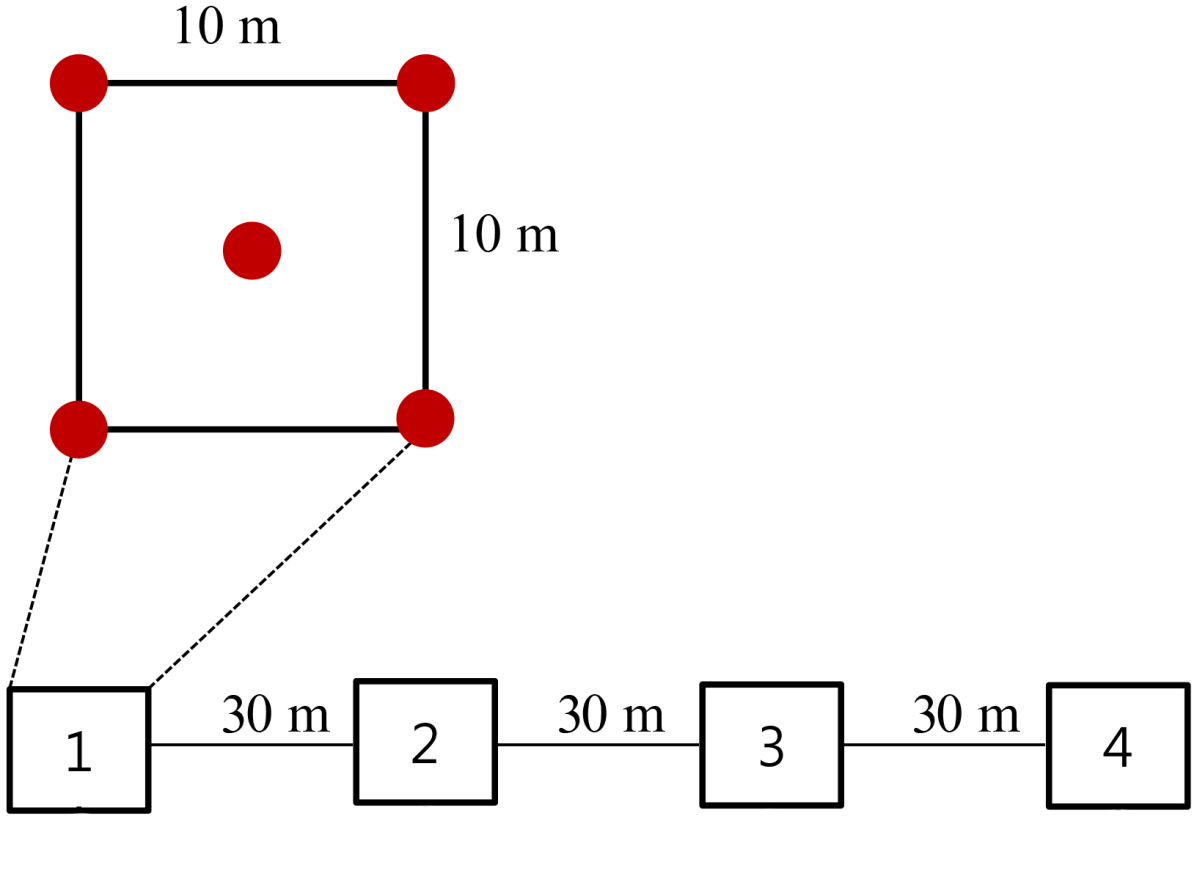


# Fig. S1


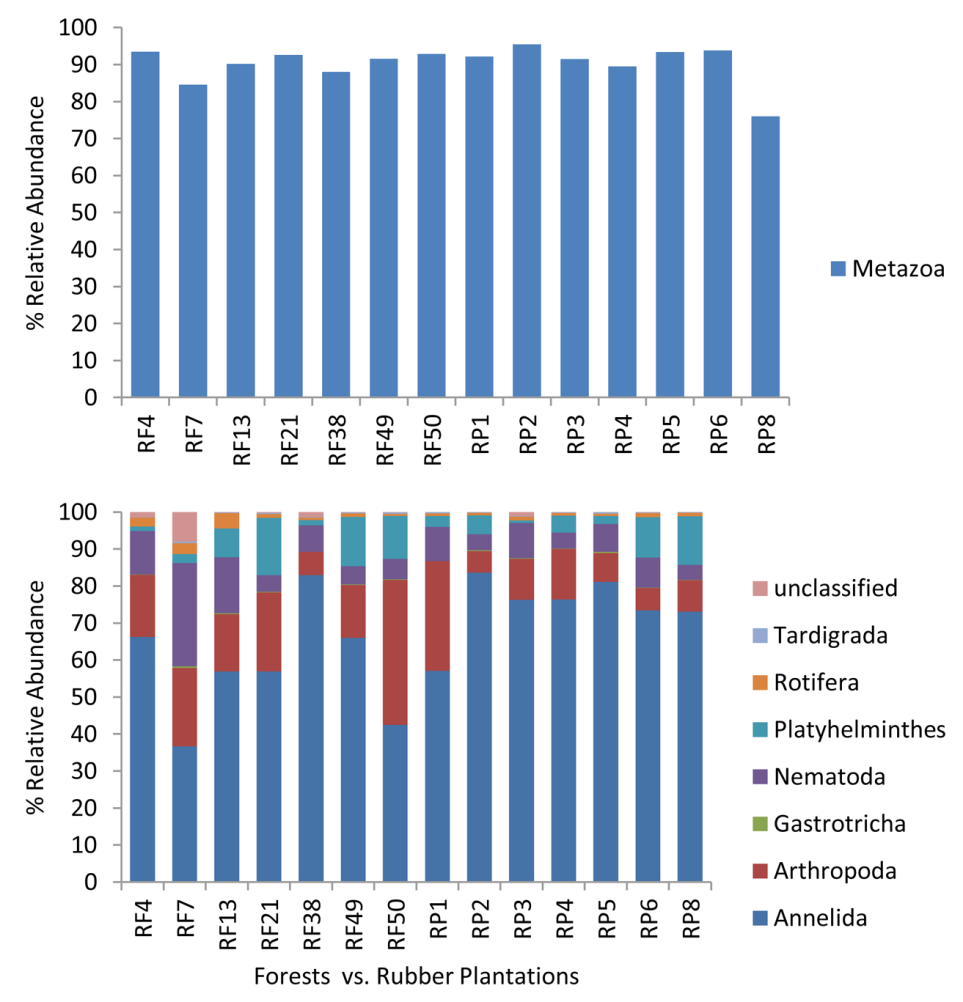


# Fig. S2


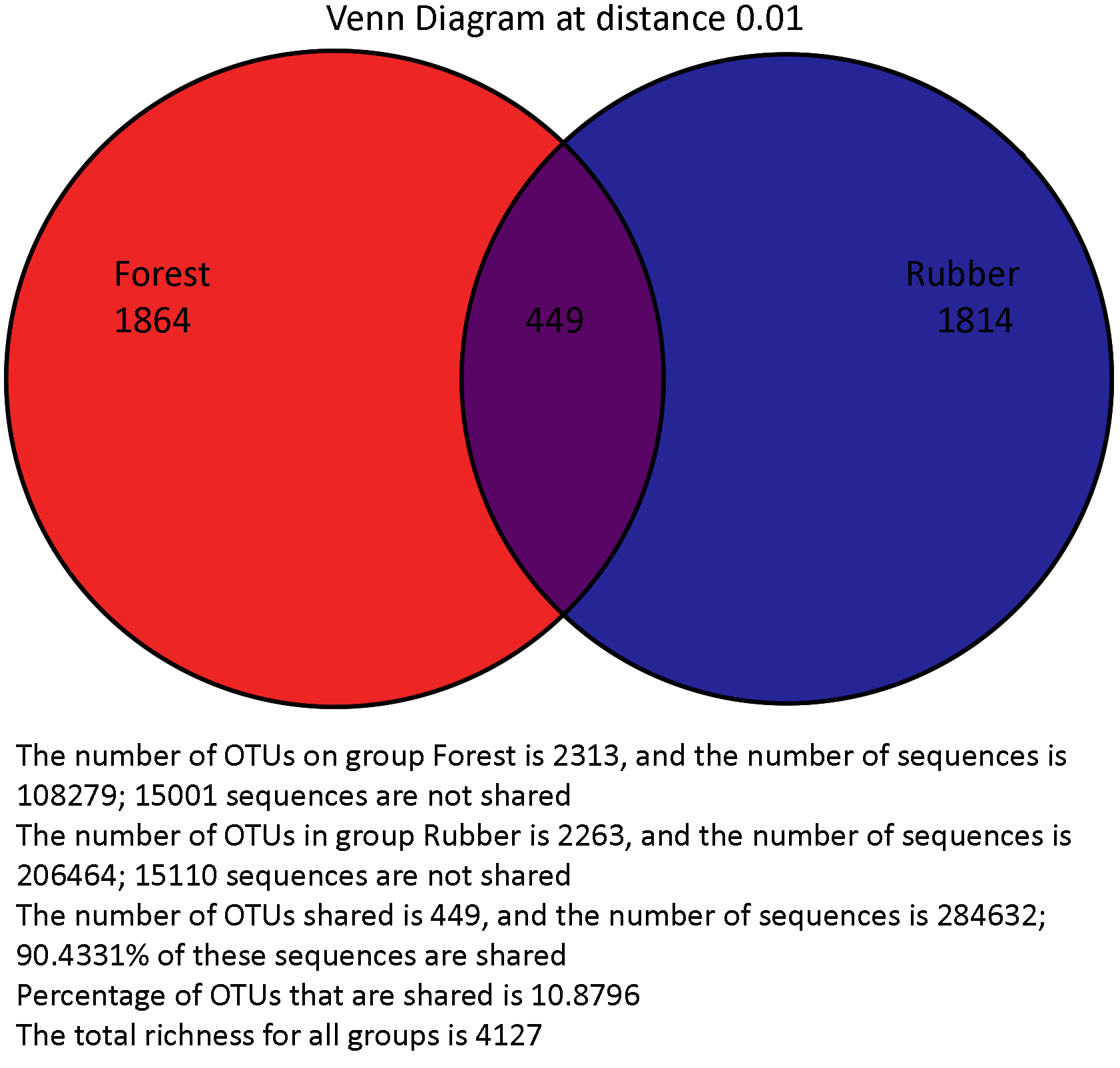


Fig. S3


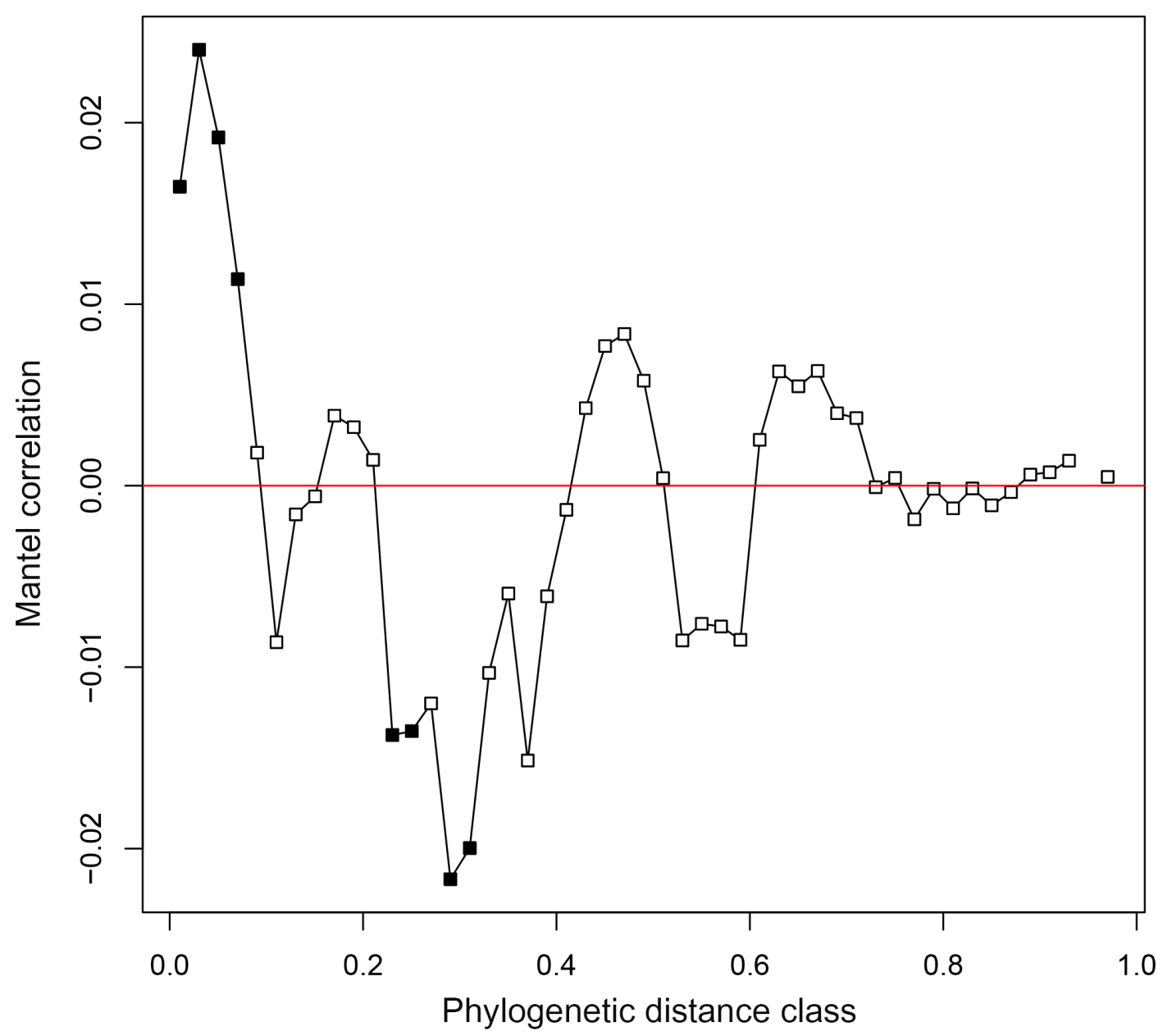


Fig. S4

#
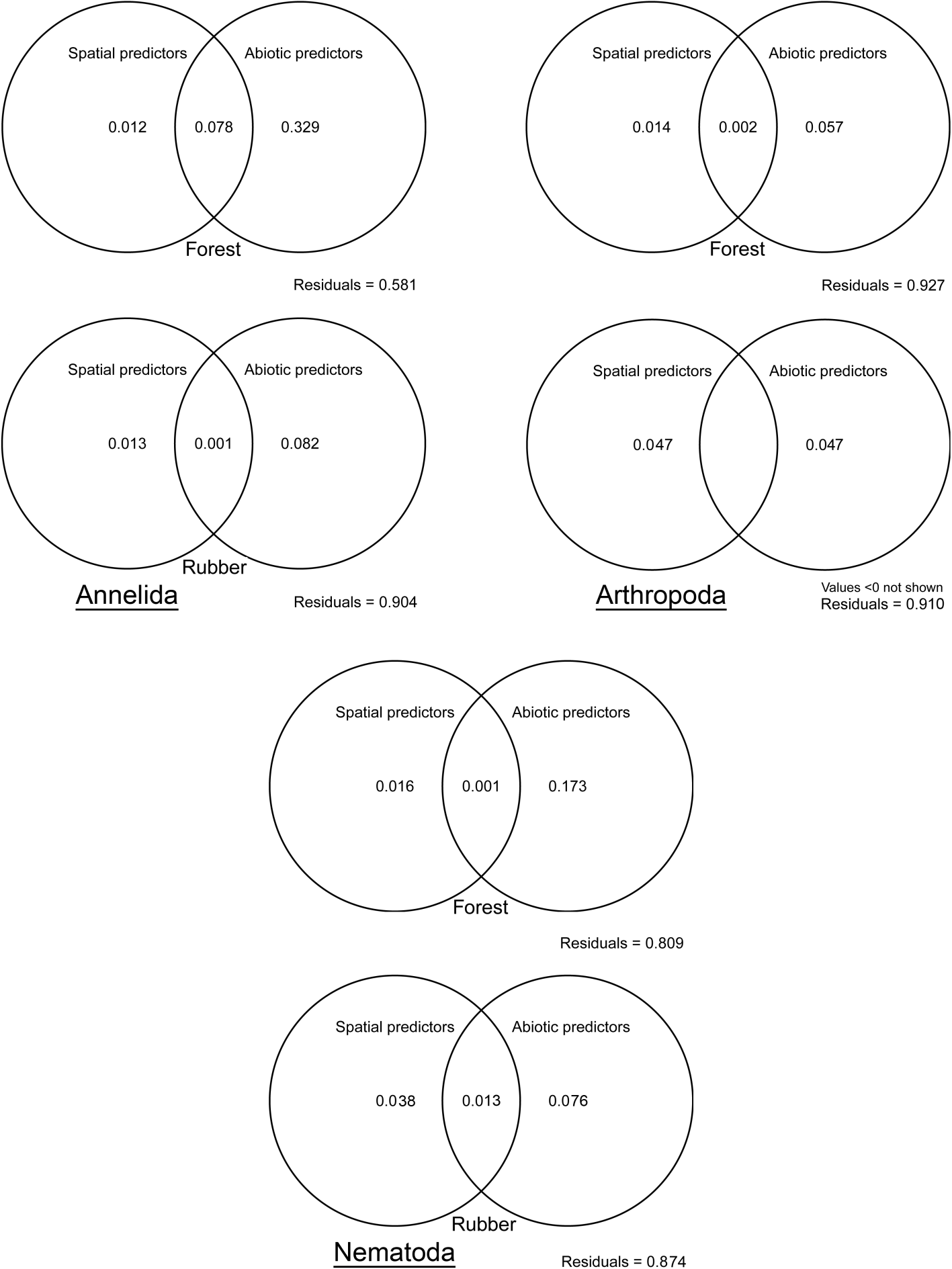


Fig. S5


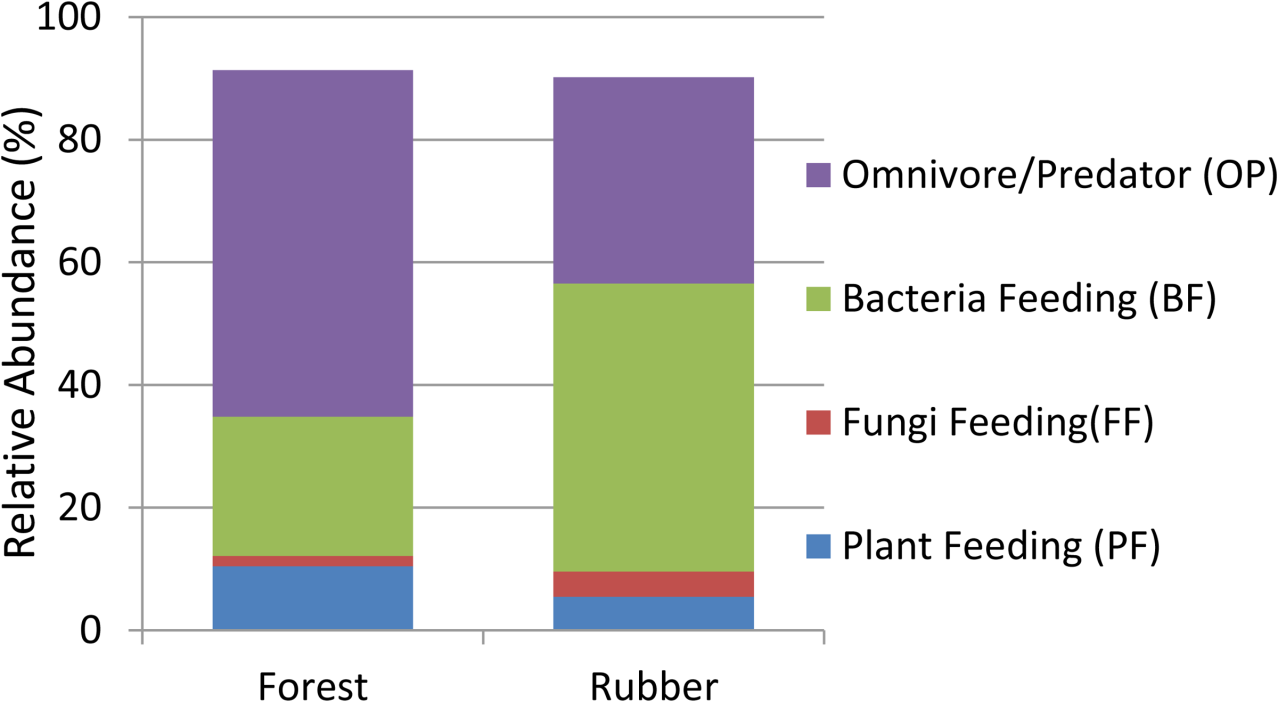


Fig. S6

# References:

1 Bonan, G., B. *Ecological Climatology: Concepts and Applications*. 2, revised edn, (Cambridge University Press, 2008).

2 Porazinska, D. L. *et al.* Evaluating high-throughput sequencing as a method for metagenomic analysis of nematode diversity. *Mol Ecol Resour* **9**, 1439-1450, doi:10.1111/j.1755-0998.2009.02611.x (2009).

3 Riaz, T. *et al.* ecoPrimers: inference of new DNA barcode markers from whole genome sequence analysis. *Nucleic Acids Res* **39**, doi:ARTN e145 10.1093/nar/gkr732 (2011).

4 Myers, E. W. & Miller, W. Optimal alignments in linear space. *Comput Appl Biosci* **4**, 11-17 (1988).

5 Schloss, P. D., Gevers, D. & Westcott, S. L. Reducing the Effects of PCR Amplification and Sequencing Artifacts on 16S rRNA-Based Studies. *Plos One* **6** (2011).
